# Prognostic value of LINE-1 methylation level in 321 patients with primary liver cancer including hepatocellular carcinoma and intrahepatic cholangiocarcinoma

**DOI:** 10.18632/oncotarget.25124

**Published:** 2018-04-17

**Authors:** Tatsunori Miyata, Yo-Ichi Yamashita, Yoshifumi Baba, Kazuto Harada, Takanobu Yamao, Naoki Umezaki, Masayo Tsukamoto, Yuki Kitano, Kensuke Yamamura, Kota Arima, Shigeki Nakagawa, Hirohisa Okabe, Katsunori Imai, Daisuke Hashimoto, Akira Chikamoto, Mototsugu Shimokawa, Hideo Baba

**Affiliations:** ^1^ Department of Gastroenterological Surgery, Graduate School of Life Sciences, Kumamoto University, Kumamoto, Japan; ^2^ Clinical Research Institute, National Kyushu Cancer Center, Fukuoka, Japan

**Keywords:** LINE-1, methylation, epigenetics, primary liver cancer, prognosis

## Abstract

**Background:**

The methylation level of long interspersed nucleotide element-1 (LINE-1) is a good surrogate marker of the global DNA methylation level. The relationship between LINE-1 methylation level and prognosis in primary liver cancer (PLC) patients remains unclear.

**Results:**

LINE-1 methylation levels were significantly lower in HCC and cHCC-CC tissues, but not in ICC tissues, than those in noncancerous liver parenchyma (HCC: *p* < 0.0001; cHCC-CC: *p <* 0.001; and ICC: *p* = 0.053). HCC cases with hypomethylated LINE-1 had significantly shorter relapse-free survival (RFS) (log-rank, *p* = 0.008); however, this was not observed for the cHCC-CC or ICC cases. Multivariate Cox regression analysis revealed a significantly higher HCC recurrence rate in the group with hypomethylated LINE-1 (hazard ratio, 1.62; 95% confidence interval, 1.06–2.58; *p* = 0.025).

**Conclusions:**

The genome-wide DNA hypomethylation status estimated via LINE-1 methylation levels might be indicative of poor RFS in patients with HCC but not ICC or cHCC-CC.

**Methods:**

We evaluated the level of LINE-1 methylation in 321 cases of curatively resected PLC {231 hepatocellular carcinoma (HCC), 19 combined hepatocellular and cholangiocarcinoma (cHCC-CC) and 71 intrahepatic cholangiocarcinoma (ICC)} via pyrosequencing of formalin-fixed paraffin-embedded (FFPE) tissues and examined its prognostic value.

## INTRODUCTION

Primary liver cancer (PLC) is the sixth most common cancer and the second leading cause of cancer death worldwide [1]. Pathologically, approximately 85–90% of PLC can be classified as hepatocellular carcinoma (HCC) and 5–10% as intrahepatic cholangiocarcinoma (ICC), with combined hepatocellular and cholangiocarcinoma (cHCC-CC) representing a small portion of PLC [2–4]. Many cases of HCC arise from cirrhosis resulting from chronic infection by hepatitis B and C virus (HBV and HCV, respectively), alcoholic injury, and non-alcoholic fatty liver disease (NAFLD), which are increasing in incidence with changes in lifestyles [5, 6]. ICC is an aggressive cancer arising from epithelial cells of the bile duct or hepatocytes [7], and its development has been associated with primary sclerosing cholangitis, hepatitis virus infection, alcohol consumption, smoking, fatty liver disease, diabetes, cholelithiasis, and choledocholithiasis. In addition, risk factors of ICC vary depending on the region [8]. cHCC-CC is currently defined as an unequivocal mixture of both HCC and ICC. According to the recent definition from the WHO, the cHCC-CC category comprises two histological forms: a classic type and a subtype with stem cell-like features [9]. All three cancers belong to the same category as PLC; however, clinically, ICC and cHCC-CC often show much more aggressive behavior with poorer prognosis than does HCC, with no standard treatment other than curative surgical resection [2–4]. Thus, the clinical features of these three types of cancer are likely distinct.

Cancer initiation and progression are caused by concurrent changes in multiple genes via genetic and epigenetic alterations leading to the activation of oncogenes or the suppression of tumor suppressor genes [10]. Along with genetic mutations, epigenetic changes such as DNA methylation and histone acetylation are important for carcinogenesis and tumor development [11, 12]. Cancer cells exhibit two types of alterations in DNA methylation: one is global DNA hypomethylation, and the other is site-specific CpG island promoter hypermethylation [13, 14]. Global DNA hypomethylation plays an important role in genomic instability, and site-specific promoter hypermethylation can silence tumor suppressor genes, leading to cancer development [15–18]. Since the long interspersed nucleotide element-1 (LINE-1) retrotransposon constitutes a substantial portion of the human genome (approximately 17%), the methylation status of LINE-1 reflects the global DNA methylation status [19]. LINE-1 hypomethylation is associated with poor prognosis in esophageal, gastric, colorectal, pancreas and breast cancer [20–24], and it can be measured in a cost-effective manner via high-throughput pyrosequencing techniques [25–27]. Therefore, the methylation level of LINE-1 may be an effective biomarker for prognostic prediction.

We previously reported that LINE-1 hypomethylation was associated with poor prognosis in 208 patients with HCC [28]; However, there are no reports that comprehensively analyzed LINE-1 methylation levels in PLCs including ICC and cHCC-CC. Therefore, the aims of this study are as follows: to confirm the association between LINE-1 methylation levels and prognosis in HCC by using a greater number of samples; to examine prognostic significance in ICC and cHCC-CC; to analyze the difference in the characteristics of LINE-1 methylation levels among the various subtypes of PLC (i.e., HCC, ICC, cHCC-CC).

## RESULTS

### LINE-1 methylation level in PLC

**Figure 1:**
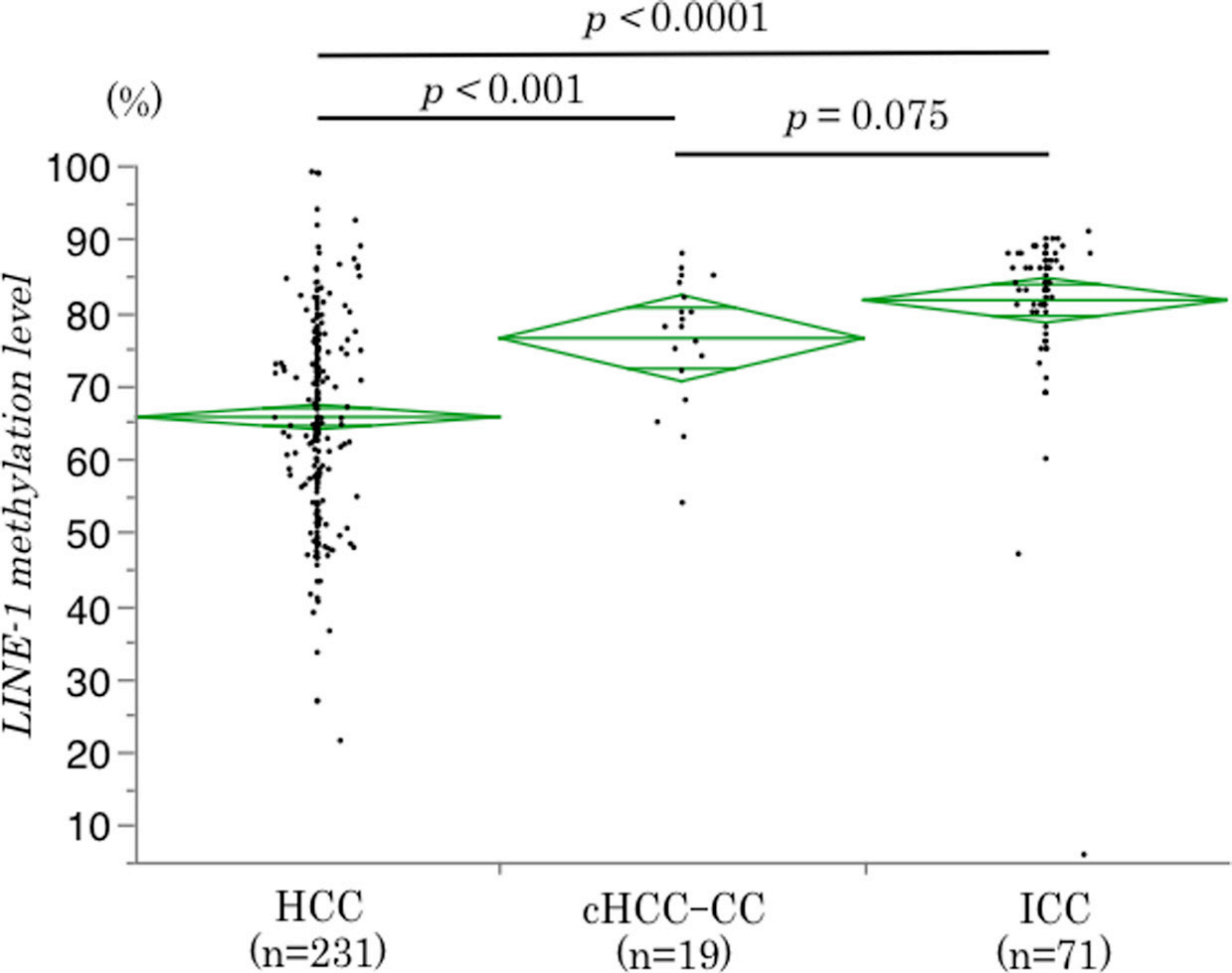
Differences in LINE-1 methylation levels among PLC subtypes LINE-1 methylation levels were different among PLC subtypes, with the lowest levels for HCC. The diamonds mean 95% confidence interval of the mean value.

We examined LINE-1 methylation levels in 321 PLC patients including 231 HCC, 19 cHCC-CC and 71 ICC cases. LINE-1 methylation levels in cancerous tissues were significantly different among the three subtypes of PLC (HCC vs cHCC-CC; *p <* 0.001, HCC vs ICC; *p <* 0.0001). HCC cases had the lowest LINE-1 methylation levels among all PLC subtypes (mean: 65.7%, range: 21.5–99.1%). However, LINE-1 methylation levels were not significantly different between cHCC-CC (mean: 76.4%, range: 54.0–88.0%) and ICC (mean: 81.6%, range: 47.0–91.0%) (*p* = 0.075) (Figure 1). For HCC, LINE-1 methylation levels were lower in the HCV-infected groups than those in the non-HCV-infected group, while similar differences were not observed for cHCC-CC or ICC (Table 1).

**Table 1:** Association between the LINE-1 methylation level in cancerous tissues and clinicopathological characteristics of 321 patients with primary liver cancer

Variables	HCC (*n* = 231)	LINE-1 methylation level (%) [mean ± SE]	*P*	cHCC-CC (*n* = 19)	LINE-1 methylation level (%) [mean ± SE]	*P*	ICC (*n* = 71)	LINE-1 methylation level (%) [mean ± SE]	*P*
Age (yrs)			0.489			0.361			0.991
≥75	39	64.3 ± 2.2		2	75.8 ± 2.2		16	81.6 ± 2.9	
<75	192	65.9 ± 1.0		17	82.0 ± 6.3		55	81.6 ± 1.6	
Sex			0.869			0.343			0.913
Male	189	65.6 ± 1.0		16	75.6 ± 2.2		22	81.8 ± 2.5	
Female	42	66.0 ± 2.1		3	81.0 ± 5.1		49	81.5 ± 1.7	
HBs Ag			0.117			0.615			0.981
Positive	66	67.9 ± 1.7		5	78.2 ± 4.0		8	81.5 ± 4.1	
Negative	164	64.7 ± 1.1		14	75.8 ± 2.4		63	81.6 ± 1.5	
HCV Ab			0.039			0.228			0.276
Positive	113	63.7 ± 1.3		7	73.1 ± 3.3		14	84.6 ± 3.1	
Negative	117	67.5 ± 1.3		12	78.3 ± 2.5		57	80.8 ± 1.5	
Child-Pugh classification			0.269			-			0.590
A	206	66.0 ± 1.0		19	76.4 ± 2.0		69	81.5 ± 1.4	
B	25	62.8 ± 2.7		0			2	86.0 ± 8.3	
ICG-R15			0.196			0.472			0.445
≥10	91	64.4 ± 1.5		8	78.1 ± 3.3		13	83.7 ± 3.4	
<10	114	66.9 ± 1.3		10	74.9 ± 2.9		51	80.8 ± 1.7	
Total bilirubin (mg/dl)			0.729			0.450			0.686
>1.0	43	66.3 ± 2.1		4	79.5 ± 4.8		10	80.2 ± 3.7	
≤1.0	188	65.5 ± 1.0		15	75.6 ± 2.3		61	81.8 ± 1.5	
Albumin (g/dl)			0.801			0.853			0.072
>3.9	143	65.5 ± 1.1		13	76.2 ± 2.5		46	76.8 ± 1.7	
≤3.9	88	66.0 ± 1.5		6	77.0 ± 3.7		25	85.0 ± 2.9	
PT (%)			0.929			0.439			0.258
≥85	175	65.7 ± 1.0		16	77.1 ± 2.2		63	81.0 ± 1.5	
<85	56	65.5 ± 1.8		3	72.7 ± 5.2		8	86.0 ± 4.1	
AFP (ng/mL)			0.488			-			-
>7.0	154	65.2 ± 1.1		-	-		-	-	
≤7.0	77	66.6 ± 1.6		-	-		-	-	
DCP (mAU/mL)			0.032			-			-
≥40	164	64.4 ± 1.1		-	-		-	-	
<40	67	68.7 ± 1.7		-	-		-	-	
CA19-9 (U/mL)			-			-			0.777
≥37	-	-		-	-		22	82.2 ± 2.5	
<37	-	-		-	-		49	81.3 ± 1.7	
Differenciation			0.358			-			0.965
Well-moderate	176	66.1 ± 1.0		-	-		54	81.4 ± 1.6	
Poor	53	64.1 ± 1.9		-	-		11	81.5 ± 3.6	
Tumor size (mm)			0.480			0.975			0.760
≥50	82	66.5 ± 1.5		8	76.5 ± 3.2		22	82.2 ± 2.5	
<50	149	65.2 ± 1.1		11	76.4 ± 2.7		49	81.3 ± 1.7	
Tumor number			0.299			0.038			0.893
Single	165	65.1 ± 1.1		13	79.2 ± 2.2		57	81.7 ± 1.6	
Multiple	66	67.1 ± 1.7		6	70.3 ± 3.3		14	81.2 ± 3.1	
Vascular invasion			0.565			0.809			0.239
Present	106	65.1 ± 1.3		13	76.8 ± 2.5		34	83.3 ± 2.0	
Absent	125	66.1 ± 1.2		6	75.7 ± 3.7		37	80.0 ± 1.9	
LN status			-			-			0.276
0	-	-		-	-		22	83.6 ± 2.5	
1	-	-		-	-		9	76.2 ± 3.9	
X	-	-		-	-		40	81.7 ± 1.8	
F stage			0.024			-			0.343
F1-3	140	67.0 ± 1.1		-	-		60	80.9 ± 1.5	
F4	80	62.7 ± 1.5		-	-		7	85.4 ± 4.5	
Procedure			0.178			0.607			0.209
Minor	172	65.0 ± 1.0		12	77.3 ± 2.6		26	83.9 ± 2.7	
Major	59	67.7 ± 1.8		7	75.0 ± 3.4		45	80.3 ± 1.7	

Abbreviations: HCC, Hepatocellular carcinoma; ICC, Intrahepatic cholangiocarcinoma; cHCC-CC, Combined hepatocellular and cholangiocarcinoma; HBs Ag, Hepatitis B surface antigen; HCV Ab, Hepatitis C virus-specific antibody; ICG-R15, Indocyanine green retention rate at 15 minutes; PT, Prothrombin time; AFP, Alpha-fetoprotein; DCP, Des-gamma-carboxy prothrombin; CA19-9: Carbohydrate antigen 19-9; LN, Lymph node.

### LINE-1 methylation level in PLC and noncancerous liver tissue

**Figure 2:**
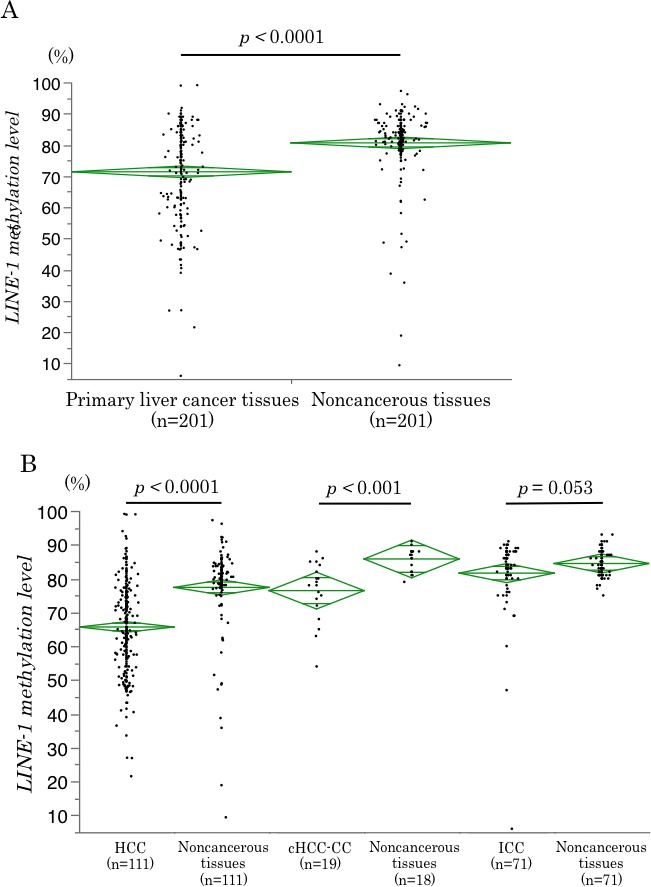
LINE-1 methylation levels in cancerous and noncancerous tissues (**A**) LINE-1 methylation levels in cancerous tissues and noncancerous liver parenchyma in 201 PLC patients. The levels were significantly lower in the cancerous tissues than in the noncancerous liver tissues (*p <* 0.0001). (**B**) LINE-1 methylation levels in cancerous tissues and noncancerous liver tissues for each histological subtype of PLC. The levels were significantly lower in the cancerous tissues than in the noncancerous liver parenchyma for HCC (*p <* 0.0001) and cHCC-CC (*p <* 0.001), but not for ICC (*p* = 0.053). The diamonds mean 95% confidence interval of the mean value.

Next, we examined LINE-1 methylation levels in 201 PLC tissues and their matched noncancerous liver parenchyma, including 111 HCC, 19 cHCC-CC and 71 ICC cases. LINE-1 methylation levels in the PLC tissues (vs. noncancerous liver parenchyma) were distributed as follows: mean 69.8 (80.6); median 72.0 (82.0); standard deviation (SD) 14.7 (10.9); and range 6–99.1 (9.3–97.3). LINE-1 methylation levels were significantly lower in PLC tissues than those in noncancerous liver parenchyma (*p <* 0.0001) (Figure 2A). In addition, LINE-1 methylation levels were significantly lower in HCC and cHCC-CC than those in their matched parenchyma (HCC: *p <* 0.0001, cHCC-CC: *p <* 0.001). In Student’s *t* test, LINE-1 methylation level was also lower in ICC; however, this difference was not statistically significant (*p* = 0.053) (Figure 2B). Similarly, in paired *t* test, there were significantly differences between cancerous tissue and noncancerous tissue in HCC (*p <* 0.0001) and cHCC-CC (*p <* 0.001), however, there was not in ICC (*p* = 0.167). These differences were also observed between PLC and noncancerous liver parenchyma irrespective of the presence or absence of hepatitis virus infection (Supplementary Figure 1). However, there was no effect of hepatitis virus infection on LINE-1 methylation level in non-cancerous part of PLC (HCC; p = 0.604, cHCC-CC; *p* = 0.373 and ICC; *p* = 0.876) (Supplementary Figure 1). Therefore, the LINE-1 methylation status between cancer and noncancerous liver parenchyma was different for HCC and cHCC-CC but not for ICC.

### Association between LINE-1 methylation level and clinical, epidemiological, and pathological variables

We next examined the relationship between LINE-1 methylation level in PLC and various clinical or pathological variables. There were significant correlations between LINE-1 methylation level and HCV-specific antibody (HCV Ab)-positivity (*p* = 0.039), des-gamma-carboxy prothrombin (DCP) (*p* = 0.032) and F stage (*p* = 0.024) in HCC and between LINE-1 methylation level and tumor number in cHCC-CC (*p* = 0.038). The other factors were not significantly different between the groups in any of the subtypes of PLC (Table 1).

### LINE-1 methylation level and patient prognosis

**Figure 3:**
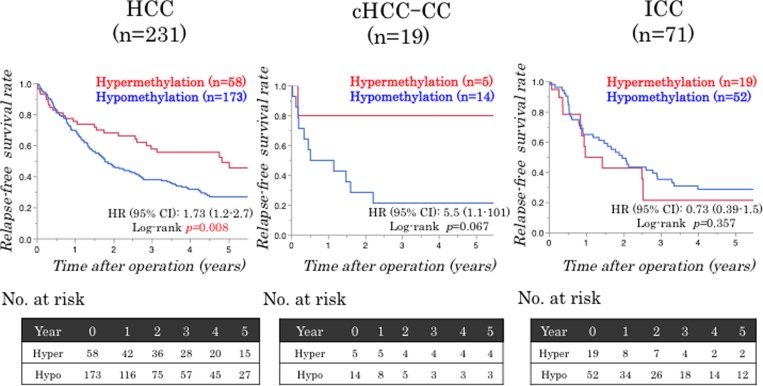
Relationships between the LINE-1 methylation status and patient survival LINE-1 hypomethylation is significantly correlated with poor RFS in HCC (*p* = 0.008) but not cHCC-CC (*p* = 0.067) or ICC (*p* = 0.357) patients.

**Figure 4:**
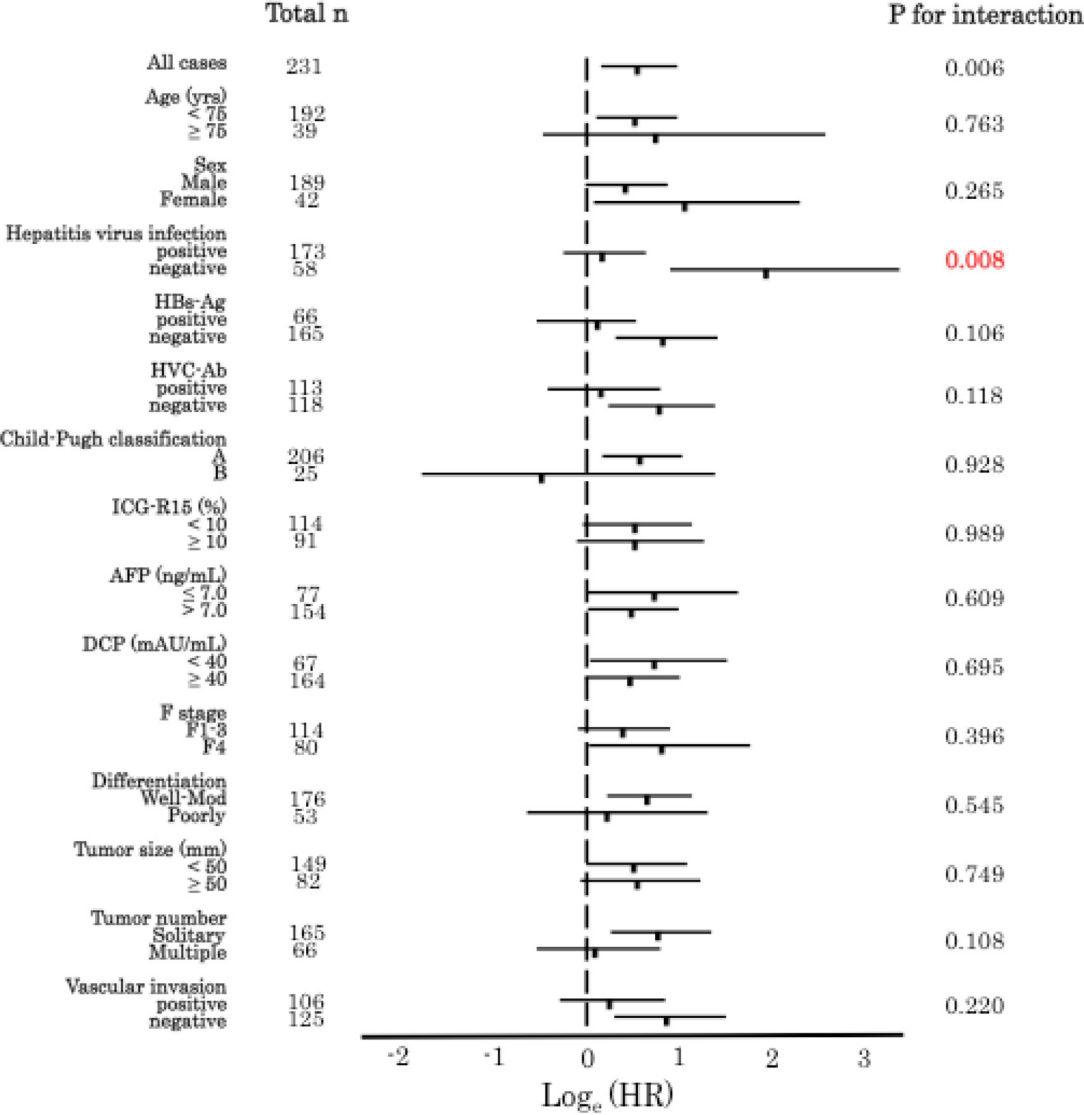
LINE-1 methylation levels in HCC and RFS in various subgroups Log_e_ (adjusted HR) plots for RFS rates in the LINE-1 hypomethylation (Q2–4) and hypermethylation (Q1) groups. Values corresponding to 95% CIs are also indicated. The relationship between LINE-1 methylation level and the RFS rate was significantly modified by hepatitis virus infection *(p* for interaction = 0.008). Bars indicated 95% confidence intervals.

A follow-up study on the 321 patients revealed 188 cancer recurrences (58.6%) and 117 deaths (36.4%). The median follow-up time for the diseased patients was 4.0 years. Using LINE-1 methylation level as a quartile categorical variable for each subtype of PLC. Using the methylation level as a quartile categorical variable (i.e. first quartile cases [Q1; ≥75.28%], second quartile cases [Q2; 65.61–75.28%], third quartile cases [Q3; 56.49–65.61%], and fourth quartile cases [Q4; < 56.49%] in HCC; [Q1; ≥ 84.0%], [Q2; 78.0–84.0%], [Q3; 72.0–78.0%] and Q4; < 72.0% in cHCC-CC; [Q1; ≥ 88.0%], [Q2; 84.0–88.0%], [Q3; 80.0–84.0%] and [Q4; <80.0%] in ICC). We adopted a dichotomous LINE-1 methylation level, defining cases in Q1 as the ‘LINE-1 hypermethylation group’ and combining cases in Q2 to Q4 into the ‘LINE-1 hypomethylation group’ for each subtype of PLC (Supplementary Table 1). Then, we analyzed the correlation between the LINE-1 methylation status and prognosis for the 321 PLC patients. Interestingly, LINE-1 hypomethylation was associated with unfavorable RFS for HCC (*p* = 0.008); however, there were no significant differences between the LINE-1 hypomethylation and hypermethylation groups for cHCC-CC (*p* = 0.067) or ICC (*p* = 0.357) (Figure 3). Multivariate Cox regression analysis also revealed that LINE-1 hypomethylation was an independent prognostic factor for HCC (HR; 1.62, 95% CI; 1.06–2.58, *p* = 0.025) (Table 2). Additionally, we examined whether the effect of LINE-1 hypomethylation on cancer recurrence was modified by any of the clinical and pathological variables, including age, sex, hepatitis virus infection (HBV or HCV), Child–Pugh classification, ICG-R15, stage of fibrosis, tumor size, number of tumors, and differentiation. The relationship between LINE-1 methylation level and RFS rate was significantly modified by hepatitis virus infection (*p* of interaction = 0.008) (Figure 4). On the other hand, in univariate and multivariate Cox regression analyses for ICC, LINE-1 hypomethylation was not an independent prognostic factor (*p* = 0.372), while vascular invasion (*p* = 0.007) and lymph node metastasis (*p* = 0.019) were independent prognostic factors (Table 2). We did not analyze the prognostic factors for cHCC-CC in RFS and OS because of the small number of cases.

**Table 2:** Univariate and multivariate analyses for RFS

Variables	HCC (*n* = 231)				ICC (*n* = 71)			
	Univariate		Multivariate		Univariate		Multivariate	
	HR (95% CI)	*P*	HR (95% CI)	*P*	HR (95% CI)	*P*	HR (95% CI)	*P*
Age ≥75 yrs vs. <75 yrs	1.11 (0.72–1.67)	0.607			1.65 (0.80–3.15)	0.165		
Male vs. Female	1.33 (0.89–2.10)	0.171			2.01 (1.09–3.59)	0.026		
Hapatitis virus infection positive vs.negative	1.24 (0.86–1.83)	0.253			0.73 (0.38–1.33)	0.305		
HBs Ag positive vs.negative	0.92 (0.64–1.32)	0.674			0.95 (0.36–2.06)	0.897		
HCV Ab positive vs.negative	1.24 (0.90–1.71)	0.189			0.67 (0.29–1.35)	0.276		
Child-Pugh classification B vs. A	1.51 (0.89–2.41)	0.122			1.44 (0.23–4.70)	0.636		
ICG-R15 ≧10 vs. <10%	1.65 (1.17–2.33)	0.005	1.58 (1.12–2.25)	0.010	0.87 (0.39–1.75)	0.720		
AFP >7.0 vs. ≤7.0 ng/mL	1.21 (0.86–1.72)	0.285			–	–		
DCP ≥40 vs. <40 mAU/mL	1.17 (0.82–1.70)	0.394			–	–		
CA19-9 ≥37 vs. <37 U/mL	–	–			1.71 (0.95–3.03)	0.075		
F stage F4 vs. F1-3	1.34 (0.96–1.88)	0.087			0.88 (0.30–2.04)	0.789		
Poor vs. well-moderate differentiation	1.03 (0.69–1.50)	0.887			1.06 (0.48–2.12)	0.870		
Tumor size ≧50 vs. <50 mm	1.33 (0.89–2.10)	0.208			2.03 (1.09–3.66)	0.026		
Multiple tumors vs. single tumor	1.24 (0.89–1.72)	<0.0001	2.25 (1.56–3.22)	<0.0001	1.33 (0.89–2.10)	0.367		
Vascular invasion present vs. absent	1.34 (0.97–1.84)	0.077			2.15 (1.22–3.85)	0.009	2.20 (1.24–3.96)	0.007
LN status pN1 vs. pN0, NX	–	–		–	2.56 (1.15–5.11)	0.023	2.66 (1.19–5.31)	0.019
LINE-1 hypomethylation vs. hypermethylation	1.73 (1.17–2.67)	0.006	1.62 (1.06–2.58)	0.025	0.73 (0.39–1.48)	0.372		

Abbreviations: HBs Ag, Hepatitis B surface antigen; HCV Ab, Hepatitis C virus-specific antibody; ICG-R15, Indocyanine green retention rate at 15 minutes; AFP, Alpha-fetoprotein; DCP, Des-gamma-carboxy prothrombin; CA19-9, Carbohydrate antigen 19-9; LN, Lymph node; HR, Hazard ratio; CI, Confidence interval.

Finally, we analyzed the relationship between OS and LINE-1 methylation level. There were no significant differences between the hyper- and hypomethylation groups in any subtype of PLC (Supplementary Figure 2). Thus, LINE-1 hypomethylation was associated with poor RFS in HCC patients only.

## DISCUSSION

To the best of our knowledge, this is the first study examining the prognostic value of LINE-1 methylation levels in PLC including HCC, ICC and cHCC-CC. Our results using 321 PLC FFPE samples showed that LINE-1 methylation levels were lower in HCC and cHCC-CC tissues but not in ICC tissues than those in the matched noncancerous liver parenchyma. This suggests that there may be differences in the epigenetic status among the subtypes of PLC. In addition, LINE-1 hypomethylation was associated with unfavorable clinical outcomes in patients with HCC only. Thus, the current study demonstrated differences in epigenetic status among PLC subtypes and the association between LINE-1 methylation and prognosis in PLC patients.

Our results suggested that global DNA hypomethylation, which leads to chromosome instability and fragility, is correlated with cancer initiation and progression in HCC and cHCC-CC but not in ICC. Udali *et al.* reported significantly lower levels of DNA methylation in HCC than those in cholangiocarcinoma (CC) tissues and comparable levels between CC and matched noncancerous liver parenchyma and gall bladder tissues [29]. Our data are consistent with their results. Thus, although HCC and ICC are similar subtypes of PLC, their epigenetic status may be distinct. One of the reasons behind the differences may be the presence of isocitrate dehydrogenase 1 (IDH1) and IDH2 mutations in ICC. IDH1 and 2 are enzymes that catalyze the oxidative decarboxylation of isocitrate to α-ketoglutarate and known as one of the driver genes of ICC [30]. IDH1/2 mutations are observed in 15 to 20% of ICC cases [31, 32] and result in the production of the 2-hydroxyglutarate (2-HG), which inhibits the , 2-HG contributes to maintaining (TET) proteins [33]. TET family enzymes (TET1, TET2, and TET3) are implicated in DNA demethylation through their dioxygenase activity that converts 5-methylcytosine to 5-hydroxymethylcytosine [34]. Thus, 2-HG contributes to maintaining DNA hypermethylation by inhibiting TET2 activity. Therefore, IDH1/2 mutations lead to DNA hypermethylation in ICC, leading LINE-1 hypermethylation. However, IDH 1/2 mutations only partly account for the differences in the methylation patterns in ICC [31, 32], and there may be other mechanisms underlying the hypermethylation of DNA in ICC compared to that in the other PLC subtypes. In addition, there was only one patient with neoadjuvant chemotherapy in ICC using FOLFOX (leucovorin and fluorouracil and oxaliplatin): the presence of preoperative chemotherapy would affect the LINE-1 methylation level in cancerous or noncancerous tissues of ICC; however, we cannot refer this issue because of the lack of samples to evaluate. Interestingly, LINE-1 methylation levels were significantly lower in cHCC-CC cancerous tissues than those in noncancerous liver parenchyma, suggesting that cHCC-CC was more similar to HCC than to ICC in terms of epigenetic status.

LINE-1 hypomethylation is associated with poor prognosis in several cancers [20–24]. The underlying mechanisms may be as follows: First, global DNA hypomethylation contributes to cancer development by inducing genomic instability [15]. Second, hypomethylation at gene regulatory regions or loss of genetic imprinting promotes the expression of oncogenes [35]. Third, LINE-1 hypomethylation activates oncogenes (e.g., c-MET [36]) and cell cycle-related genes (e.g., CDK6 [28]), which are associated with cancer progression [37]. Therefore, LINE-1 hypomethylation may also be associated with cancer progression. However, we found no association between LINE-1 hypomethylation and OS in any of the three subtypes of PLC. The present study showed that LINE-1 hypomethylation is related to short RFS in HCC patients only. We can explain this discrepancy as follows: First, re-resection for recurrent HCC can prolong OS [38, 39]. Therefore, although the RFS for HCC was short in the LINE-1 hypomethylation group, OS was not. Second, the sample size for cHCC-CC was very small. However, we could observe a trend toward poorer RFS and OS in the LINE-1 hypomethylation group than those in the LINE-1 hypermethylation group for cHCC-CC. Therefore, we need additional studies with larger sample sizes to determine whether LINE-1 hypomethylation is indicative of poor prognosis in cHCC-CC. Third, for ICC, there was no significant difference in LINE-1 methylation levels between the cancerous and noncancerous tissues, and many ICC samples exhibited high LINE-1 methylation levels. Therefore, LINE-1 methylation levels did not influence patient prognoses in ICC. Thus, further larger studies are also required for ICC.

Hepatitis virus infection is a strong prognostic factor of PLC. In the present study, the presence or absence of HCV infection had significant effect on the LINE-1 methylation level of patients with HCC (Table 1). Nishida *et al.* reported the effect of hepatitis virus infection on epigenetic status in liver diseases [40]. On the other hand, subgroup analysis showed that the risk of recurrence assessed based on LINE-1 hypomethylation was higher in the non-hepatitis virus-infected group than that in the infected group (Figure 4). Therefore, especially in patients without hepatitis virus infection, LINE-1 hypomethylation may be an effective biomarker of HCC recurrence. In the current study, comparison of primary tumor and metastatic lesion of LINE-1 methylation level had not been conducted. As Murata *et al.* reported in colon cancer [41], it can be expected that primary tumor and metastatic lesion are equivalent LINE-1 methylation in HCC. Furthermore, as you know, there are “metastasis” and “multicentric recurrence” in the recurrence pattern of HCC. The methylation level of LINE-1 in cancerous tissue may be useful for this diagnosis, therefore, it may be worthwhile to collect many clinical samples of recurrent resections of HCC.

Table 1 also showed that F4 of noncancerous lesion or higher DCP were significantly associated with lower LINE-1 methylation of HCC. In our own series, there was no statistically difference in the LINE-1 methylation level between F1-3 and F4 of noncancerous lesions (data not shown). There is a possibility that tumor environments such as F4 would affect the LINE-1 methylation level of HCC; however further *in vitro* study would need to confirm this phenomenon. As for DCP, we could not found any papers which refer the association between the higher DCP and the lower LINE-1 methylation level of HCC. DCP is well known to be a novel biomarker of malignant behavior of HCC; therefore, the association between DCP and LINE-1 methylation would be the important theme to investigate.

There are some limitations in this study, such as the small number of cHCC-CC and ICC cases. The incidence of cHCC-CC and ICC has been reported to vary from fewer than 1% to approximately 5–10% of PLC cases. Therefore, a multicenter study with a large number of cHCC-CC or ICC cases, such as a nationwide surveillance study, may be required.

In summary, we evaluated LINE-1 methylation levels using pyrosequencing and examined the prognostic values of LINE-1 hypomethylation in 321 patients with PLC. First, we could confirm prognostic significance of LINE-1 hypomethylation in HCC by using a greater number of cases. Second, LINE-1 hypomethylation was found to be not associated with poor prognosis in ICC and cHCC-CC patients. Even in the same primary liver cancer, LINE-1 methylation statuses were different. The mechanisms of epigenetic regulation in PLC and its relationship with cancer initiation and progression should be explored.

## MATERIALS AND METHODS

### Study subjects

HCC, cHCC-CC and ICC samples were collected from patients who had undergone surgical resection as their first therapy at Kumamoto University Hospital (Kumamoto, Japan) between January 2000 and February 2016. Three hundred and twenty-one patients were finally included in the current study. Patients were followed up at 1- to 3-month intervals until death or until July 1, 2017, whichever came first. Relapse-free survival (RFS) was defined as the duration between the operation and the date when cancer recurrence was observed. Overall survival (OS) was defined as the duration between the operation and the date of death. Written informed consent was obtained from each patient, and the study procedures were approved by the institutional review board.

### DNA extraction

Several 10-µm-thick sections of formalin-fixed paraffin-embedded (FFPE) tumor tissues were stained with hematoxylin and eosin (H&E), and the tumor slides were reviewed by a pathologist who marked the areas corresponding to the tumor and noncancerous liver parenchyma. Cancer tissues without stromal areas were also marked. In each case, as much of the cancer tissues without stroma as possible were macroscopically scraped off from the slides as previously described [17, 21–24, 28, 42]. The section area depended on the size of the tissue (Supplementary Figure 3). Then, DNA was extracted using a QIAamp DNA FFPE Tissue Kit (Qiagen, Valencia, CA, USA).

### Sodium bisulfite treatment and pyrosequencing for LINE-1 methylation assessment

Genomic DNA was treated with sodium bisulfite using an EpiTect Bisulfite Kit (Qiagen) as previously described [17, 21–24, 28, 42]. Polymerase chain reaction (PCR) and subsequent pyrosequencing of LINE-1 were performed as previously described using the PyroMark kit (Qiagen) [17, 21–24, 28, 42]. This assay amplifies a region of LINE-1 containing four CpG sites (base positions 305–331 in Accession No. X58075). In each tumor sample, the overall LINE-1 methylation level was determined based on the average relative amount of cytosine (C) residues among the four CpG sites (Supplementary Figure 4). Since several reports have shown that hepatocytes can differentiate into cholangiocytes, which can give rise to cHCC-CC and ICC [7, 43, 44], we considered LINE-1 methylation levels in the liver parenchyma in the calculation of overall LINE-1 methylation levels in noncancerous tissues, as well as in cHCC-CC and ICC tissues.

### Statistical analysis

The clinicopathological characteristics were summarized using descriptive statistics. We compared the means using Student’s *t* test and paired *t* test and analysis of variance between the two groups. We staged LN status using the seventh edition of the American Joint Committee on Cancer (AJCC)/International Union Against Cancer (UICC) staging manual [45] and F stage using New Inuyama classification [46]. The survival curves were estimated using the Kaplan–Meier method and compared using the log-rank test. Hazard ratios (HRs) and 95% confidence intervals (CIs) were calculated using Cox proportional hazard models. The prognostic factors for RFS and OS in HCC and ICC patients were assessed using the Cox proportional hazard models with the backward elimination method. In addition, to assess interactions among variables, the LINE-1 methylation level was cross-correlated with another variable of interest via univariate Cox modeling in HCC, and the interaction was evaluated using the Wald test. All *p*-values were two-sided, and values lower than 0.05 were considered statistically significant. JMP (version 12, SAS Institute, Cary, NC, USA) was used for statistical analyses.

## SUPPLEMENTARY MATERIALS FIGURES AND TABLE



## Abbreviations

PLC: Primary liver cancer
HCC: Hepatocellular carcinoma
ICC: Intrahepatic cholangiocarcinoma
cHCC-CC: combined hepatocellular and cholangiocarcinoma
LINE-1: Long interspersed element-1
HBV: hepatitis B virus
HCV: hepatitis C virus
NAFLD: non-alcoholic fatty liver disease
RFS: Relapse-free survival
OS: Overall survival
FFPE: formalin-fixed paraffin-embedded
H&E: hematoxylin and eosin
SD: Standard deviation
DCP: Des-gamma-carboxy prothrombin
IDH: isocitrate dehydrogenase
TET: Ten-eleven translocation
