# Supplementary material for: Prognostic value of LINE-1 methylation level in 321 patients with primary liver cancer including hepatocellular carcinoma and intrahepatic cholangiocarcinoma
Source: Oncotarget. 2018 Apr 17;9(29):20795–806. doi: 10.18632/oncotarget.25124 (PMC5945516; doi:10.18632/oncotarget.25124)
Supplement: Supplementary file 1 [file oncotarget-09-20795-s001.pdf]

Prognostic value of LINE-1 methylation level in 321 patients with primary liver cancer including hepatocellular carcinoma and intrahepatic cholangiocarcinoma

SUPPLEMENTARY MATERIALS

Supplementary Table 1: Quartile groups corresponding to LINE-1 methylation level for each subtype of PLC (*n* = 321)

	Q1	Q2	Q3	Q4
HCC (*n* = 231)	≥75.28%<br>*n* = 58	<75.28%<br>≥65.61%<br>*n* = 58	<65.61%<br>≥56.49%<br>*n* = 58	<56.49%<br>*n* = 57
cHCC-CC (*n* = 19)	≥84.0%<br>*n* = 5	<84.0%<br>≥78.0%<br>*n* = 6	<78.0%<br>≥72.0%<br>*n* = 4	<72.0%<br>*n* = 4
ICC (*n* = 71)	≥88.0%<br>*n* = 19	<88.0%<br>≥84.0%<br>*n* = 19	<84.0%<br>≥80.0%<br>*n* = 19	<80.0%<br>*n* = 14

Abbreviations: HCC, Hepatocellular carcinoma; cHCC-CC, Combined hepatocellular andcholangiocarcinoma; ICC, Intrahepatic cholangiocarcinoma.

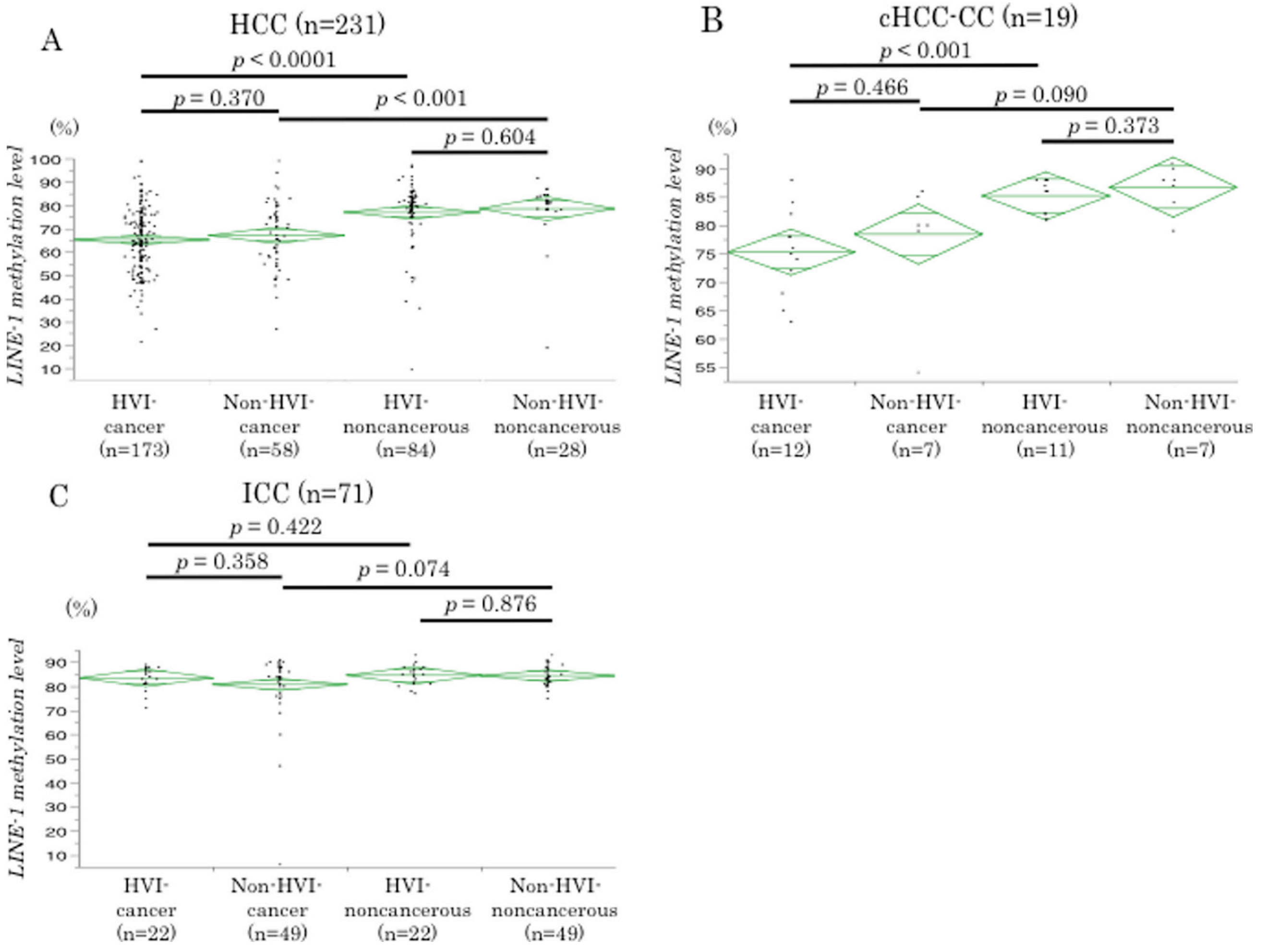

Supplementary Figure 1: LINE-1 methylation levels between PLC and noncancerous liver parenchyma with or without hepatitis virus infection. (**A**) Irrespective of hepatitis virus infection (HVI), LINE-1 methylation levels in HCC were lower in cancerous tissues than in the matched noncancerous liver parenchyma. (**B**, **C**) The same trend was observed for cHCC-CC; however, for ICC, there were no significant differences between the cancerous tissues and noncancerous liver parenchyma with or without HVI.

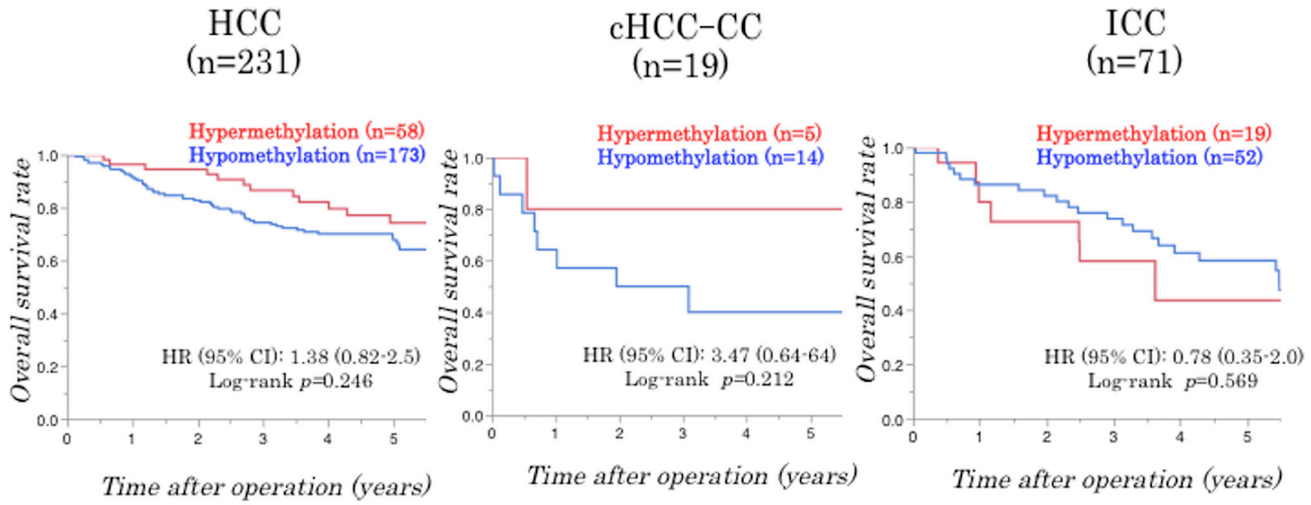

No. at risk

Year	0	1	2	3	4	5
Hyper	58	54	50	43	34	27
Hypo	173	150	133	111	91	60

No. at risk

Year	0	1	2	3	4	5
Hyper	5	4	4	4	4	4
Hypo	14	10	7	6	5	5

No. at risk

Year	0	1	2	3	4	5
Hyper	19	12	11	8	3	3
Hypo	52	44	41	34	23	20

Supplementary Figure 2: LINE-1 methylation level and OS in patients with PLC. LINE-1 hypomethylation is not significantly correlated with poor OS in any of the PLC subtypes (HCC: $p = 0.246$; cHCC-CC: $p = 0.212$; and ICC: $p = 0.569$).

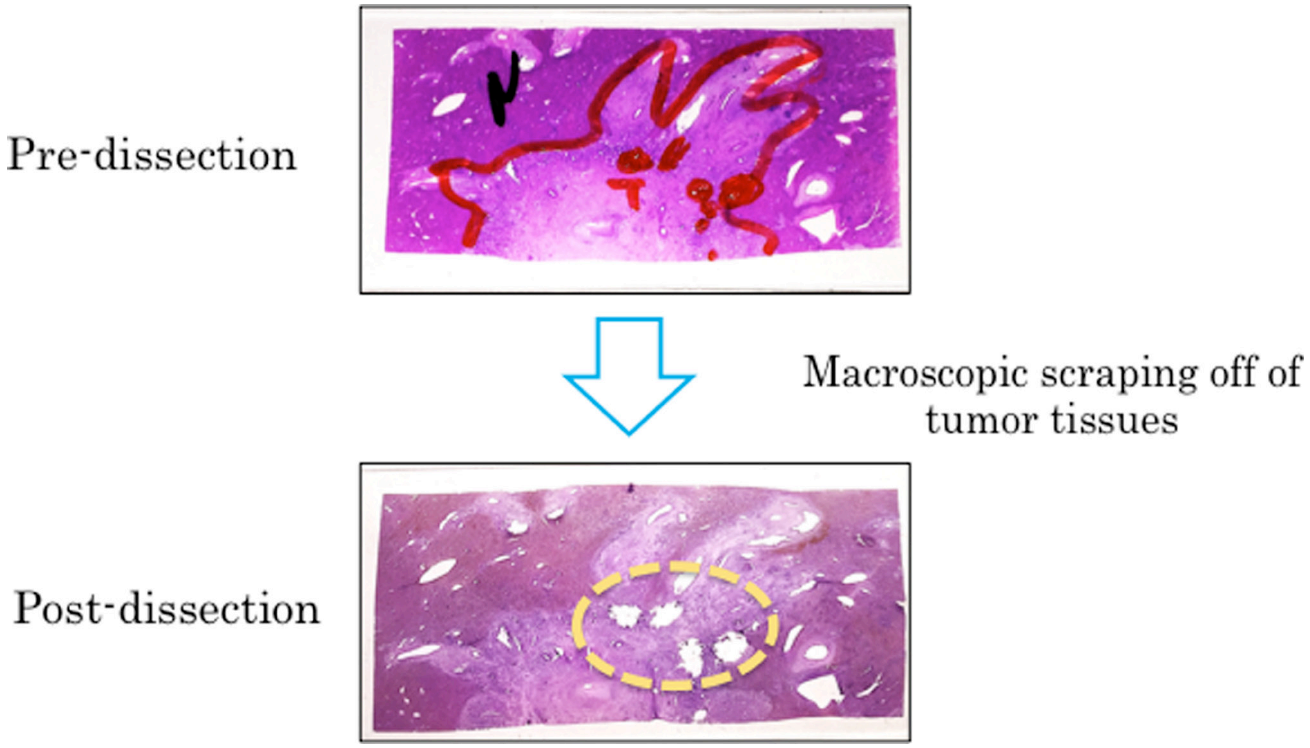

Supplementary Figure 3: Details of DNA extraction from FFPE samples. We scraped off demarcated cancerous tissues from H&E-stained slides and extracted DNA using a QIAamp DNA FFPE Tissue Kit (Qiagen, Valencia, CA, USA).

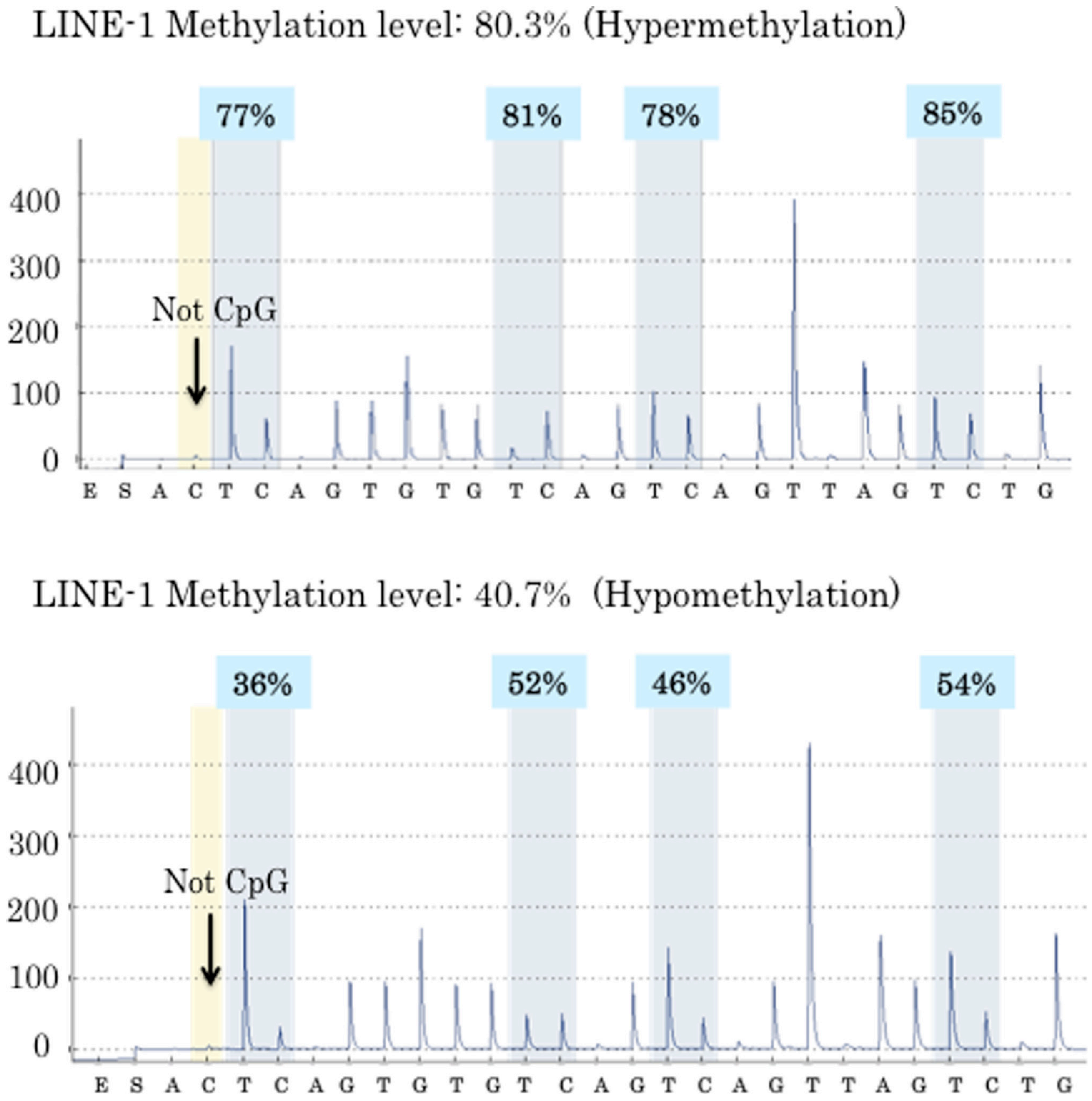

Supplementary Figure 4: LINE-1 methylation level in cancerous tissues by pyrosequencing techniques. Percentage (blue bars) indicating the proportion of cytosine (C) nucleotides at each CpG site after bisulfite conversion. From these proportions, we can measure the methylation levels at these four sites. Overall, LINE-1 methylation level is calculated as the average proportion of C residues (%) at the four CpG sites. Arrow indicating the absence of residual C residues at non-CpG sites, representing complete bisulfite conversion.
